# Challenging the diagnosis of Cystic Fibrosis in a patient carrying the 186-8T/C allelic variant in the CF Transmembrane Conductance Regulator gene

**DOI:** 10.1186/1471-2466-14-44

**Published:** 2014-03-13

**Authors:** Sara Caldrer, Genny Verzè, Jan Johansson, Claudio Sorio, Chiara Angiari, Mario Buffelli, Baroukh Maurice Assael, Paola Melotti

**Affiliations:** 1 Department of Pathology and Diagnostics, General Pathology Section, University of Verona School of Medicine, Verona, Italy; 2 Cystic Fibrosis Center, Azienda Ospedaliera di Verona, Verona, Italy; 3 Department of Neurological Sciences, Physiology Section, University of Verona School of Medicine, Verona, Italy

**Keywords:** Cystic Fibrosis, Cystic Fibrosis Conductance Transmembrane Regulator, Mutation analysis, Nasal potential difference measurements, Cystic fibrosis conductance transmembrane regulator function

## Abstract

**Background:**

This report describe for the first time a clinical case with a CFTR allelic variant 186-8T/C (c.54-8 T/C) in intron 1 of CFTR and underline the importance of applying a combination of genetic and functional tests to establish or exclude a diagnosis of Cystic Fibrosis. In this case the diagnostic algorithm proposed for CF has been successfully applied at our Center and previous CF diagnosis assigned in a different Center was not confirmed.

**Case presentation:**

A 38 year-old Italian woman had been treated as affected by CF since 2010, following diagnosis based on sweat tests (reported values of 73 and 57 mEq/L) and a clinical history consistent with CF. No mutations were identified by first level of genetic analysis. Afterwards the patient referred to our center for assessing the relevance of these findings. The genetic variant 186-8T/C (c.54-8 T/C) in intron 1 of the CFTR gene was detected by sequencing. Low-level interstitial-alveolar infiltration was recorded by high-resolution computerized tomography. Lung function was normal and sputum and Broncho Alveolar Lavage cultures resulted bacteriologically negative. Sweat chloride levels was re-assessed and resulted with values of 57 and 35 mEq/L, with a borderline range between 40 and 60 mEq/L. Nasal Potential Difference measurements resulted in three reliable measurements consistent with a non-CF phenotype. Differential diagnosis with ciliary dyskinesia was excluded, as was exon 2 skipping of CFTR gene that might have caused a CFTR functional defect. Furthermore, single cell fluorescence analysis in response to cAMP agonists performed in patient’s monocytes overlapped those obtained in healthy donors.

**Conclusion:**

We concluded that this patient was not affected by CF. This case highlights the need for referrals to highly specialized centers and the importance of combined functional and genetic tests in making a correct diagnosis. Moreover, we confirmed a correlation between NPD tracings and cell depolarization in monocytes providing a rationale for proposing the use of leukocytes as a potential support for CF diagnosis.

## Background

Cystic Fibrosis (CF) is the most commonly occurring severe disease with autosomal recessive inheritance in Caucasians, with an incidence of approximately 1 in 3000, and among sufferers clinical manifestations vary considerably. Mutations in the CF Transmembrane Conductance Regulator (CFTR) gene bring about the disease. Up to now around 1900 mutations in the CFTR gene have been identified (http://www.genet.sickkids.on.ca). Most of them are somewhat rare and this means that genetic test results are not always sufficient for CF diagnosis [1] and, in addition, a single genotype may result in different clinical symptoms. CF is usually associated with exocrine pancreatic insufficiency, gradual loss of pulmonary function and lung tissue destruction, but it can also be associated with sufficient residual pancreatic function and a much milder clinical picture [2]. CFTR related disorders [3] include a range of mild conditions caused by CFTR dysfunction, associated with limited deterioration over time and clinical manifestations in at least one organ in the presence of normal or borderline sweat chloride values. The borderline range is between 60 and 40 or 30 mmol/L depending on age and international guidelines [4,5] with the lowest threshold for normal values only during the first year of age [4,5] or always according to De Boeck et al. [4,5]. Sometimes CF diagnosis is challenging and requires, in addition to sweat test and genetic analysis, the use of sophisticated diagnostic tools [6,7]. They aim at testing CFTR function as ion channel *in vivo* or *ex vivo* as under the inferior turbinate of the nose or in rectal biopsies by Nasal Potential Difference (NPD) measurements or intestinal current measurements (ICM), respectively. Both these tests need standardization and validation, and recently European Standard Operative Procedures have been developed by the European Cystic Fibrosis Society Diagnostic Network Working Group for use as a diagnostic aid. Other methods measuring *ex vivo* the functional properties of CFTR in patients’ tissues utilizing nasal epithelium [8], leukocytes [9] or intestinal organoids [10] have been proposed.

## Case presentation

**Figure 1:**
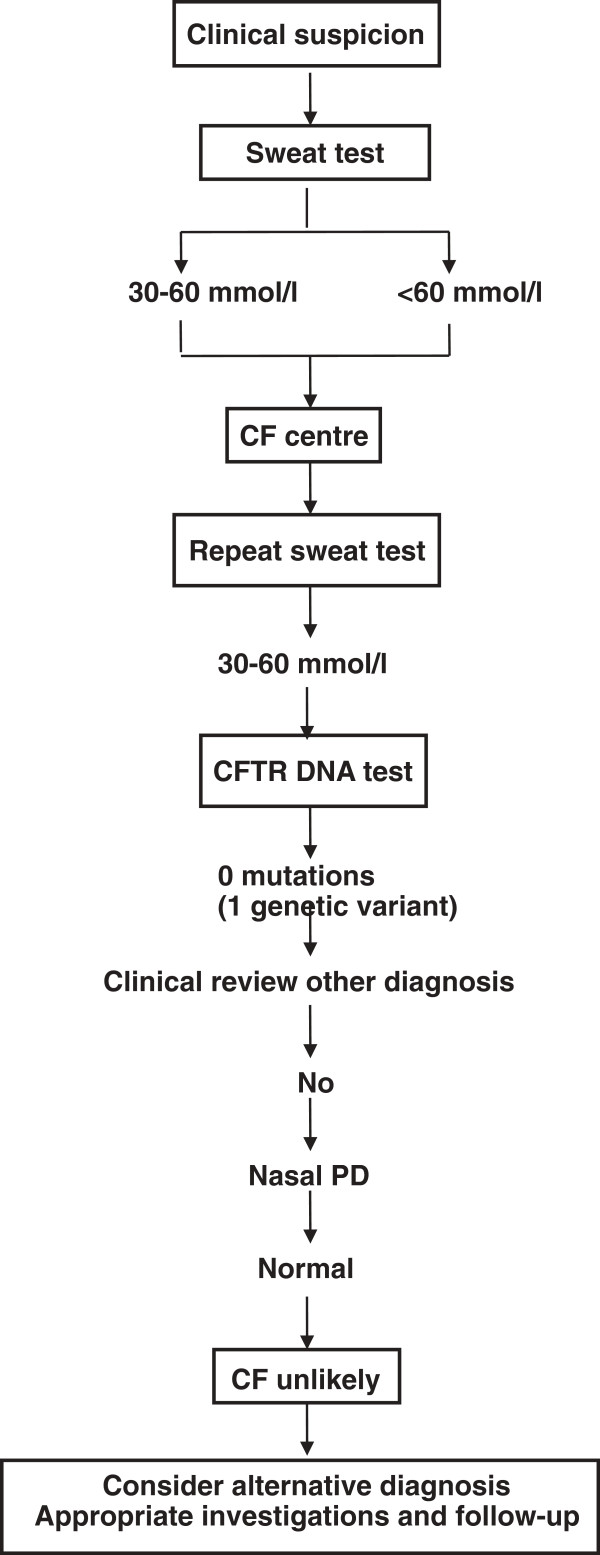
**Diagnostic algorithm: diagram describing the diagnostic algorithm utilized for the case described and based on the proposals from De Boeck et al. **[4]**.**

Here we report the clinical case of a 38 year-old Italian woman who, since 2010, had been treated as affected by CF in a European CF Center. This had followed diagnosis based on repeated sweat tests (chloride 60 and 74 mEq/L, normal values <50 mEq/L) and a clinical history consistent with CF (chronic sinusitis, recurrent bronchitis, gallstones). Chronic sinusitis was associated with deviation of the nasal septum treated surgically. Low-level infiltrative interstitial lung damage was evident from High Resolution Computerized Tomography (HRCT), bronchiectasis were absent. No mutations were identified by genetic analysis performed where the patient was diagnosed as CF. Subsequently the CFTR allelic variant 186-8T/C (c.54-8 T/C) in intron 1 of CFTR gene was identified by gene sequencing in an Italian CF Center where genetic analysis was performed by reverse dot blot (INNO-LIPA CFTR19, CFTR17 + TN Update, CFTR Italian Regional), sequencing and Multiplex ligation-dependent probe amplification (MLPA). To our knowledge, this is the first time this allelic variant has been reported. The patient was then referred to our center for assessment of the relevance of these findings. We then applied a diagnostic algorithm (Figure 1) based on established guidelines [4], where it is advised to continue the diagnostic work up if symptoms in a patient persist, as well as when symptoms have resolved but are highly suspicious for CF such as pancreatitis or Pseudomonas aeruginosa-associated lung disease. Wherever the algorithm ends with “CF unlikely” it is advised to investigate for alternative diagnoses such as primary ciliary dyskinesia, humoral immunodeficiency, Shwachman syndrome. For patients with CFTR dysfunction, the physician needs to decide the most appropriate diagnostic label (non-classic CF or other WHO diagnostic definitions in patients with very limited symptoms). Patients with a borderline sweat test (30–60 mmol/l), only one CFTR mutation identified, and an inconclusive nasal potential difference (PD) cannot at present be classified correctly. They are at least CF carriers. In the presence of persistent symptoms they need structured follow up at an appropriate facility (for some patients this may be the CF centre) and symptomatic treatment. Genetic counseling is important in these patients and their families. CFTR DNA test: screening test to search for the most frequent mutations in the population from which the patient originates. Mutation scanning of CFTR gene: this test is only necessary in some patients in whom the diagnosis cannot be supported by other means. The tests in the grey area are optional because two clearly positive sweat tests are sufficient to support the diagnosis of CF in a compatible clinical setting. However, in most CF centres the CFTR DNA test will be performed to confirm the diagnosis, to allow for further cascade screening if necessary, and at times for research purposes. Consult genetic lab: in patients with an elevated sweat chloride level it would be unusual but not impossible not to find any mutation. In case of doubt about the diagnosis, a mutation scanning of the complete gene can be done. A falsely positive sweat test and the possibility of CF heterogeneity also need to be considered.

**Figure 2:**
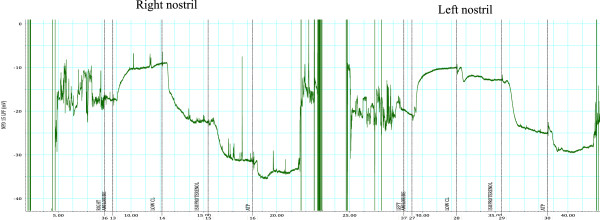
**NDP measurements.** The agents indicated at the bottom were added at the times (minutes) shown on the x-axis. On the y-axis, PD is expressed in mV. The graph on the left was obtained from right nostril measurements; the right one was obtained from left nostril measurements. Baseline NPD value was -21 mV. After the addition of amiloride PD increased to 13 mV, falling to 2.8 mV after exposure to 0 Cl- and isoproterenol. NDP measurements were repeated three times and readings were always consistent with a non-CF phenotype.

In the case here presented NPD was measured in accordance with a standardized protocol [6] and normal tracings were obtained. Three repeated measurements were consistent with a non-CF phenotype. The Wilschanski index [7] was calculated and results ranged from 0.06 to 0.24, with non-CF values < 0.82 in our Center (Figure 2). Two consecutive sweat tests were carried out 3 months apart (on May and July) in accordance with the Gibson and Cooke method [11] and chloride values of 35 mEq/L and 57 mEq/L were obtained respectively. The previously reported sweat test was performed only in the summer following an unspecified method with a local cut-off for normal value. These might be reasons for discrepancies with test results from our Center, along with patient’s diet and hydration levels. It is known that Normal and borderline sweat tests were obtained in CF patients in the presence of specific CFTR genotypes: the most representative includes mutations 3849 + 10 kb C > T, D1152H and R117H [12,13]. Lung function was normal (forced vital capacity and forced expiratory volume in the first second were both 103% and forced expiratory flow at 25-75% was 113% of predicted values) and lung infection was excluded by several sputum cultures and Broncho Alveolar Lavage (BAL). We have no data available about eventual differential cell count in BALFs. However normal pulmonary function tests and negative sputum/BAL cultures are reported in some CF patients.

We then considered primary ciliary dyskinesia as a possible, alternative diagnosis. However televideo microscopic technique carried out in a specialized center displayed normal ciliary beat and the saccharin test showed normal ciliary mucus transport, allowing the exclusion of this disease.

**Figure 3:**
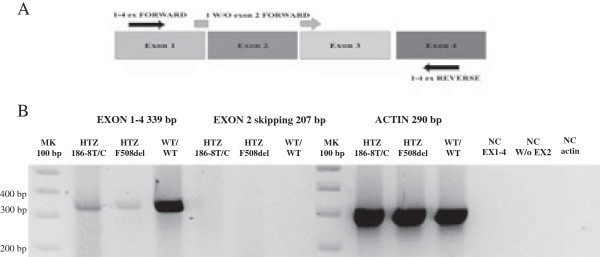
**Search for CFTR exon 2 skipping by reverse-transcriptase PCR amplification of mRNA from monocytes. (A)***RT-PCR design*: mRNA from monocytes was analyzed by RT-PCR using primers designed to amplify fragments of different length, depending on the presence or absence of exon 2, to better define if the genetic variant 186-8T/C had caused alternative splicing in this subject. Total RNA was purified and reverse-transcribed with random primers. PCR was then carried out for 35 cycles (30” at 94°C, 30” at 60°C, 30” at 72°C) using the forward primer, Ex 1–4 Forward 5’-AAAGGCCAGCGTTGTCT-3’ (black arrow) or W/O exon 2 Forward primer 5’-ACTTTTTTTCAGAGAATGGGATAGAG-3’ (gray arrow), and the reverse primer, Exon 1–4 5’-GCGATAGAGCGTTCCTC-3’ (black arrow). The exon-spanning primer “W/O exon 2 Forward primer” is localized between the exons 1 and 3 coding sequence, and thus works properly only in the absence of exon 2. Amplified fragment lengths using Exon1-4 Forward and Reverse primers (black arrows) were expected to be 339 bp. Therefore, in the case of exon 2 skipping, we would expect a shorter PCR-product (228 bp). With the exon-spanning Forward primer (W/O Exon 2; grey arrow) we expected an amplified product of a length of 207 bp. **(B)***PCR-amplification products*: We compared the PCR-amplified products obtained from the patient (186–8 T/C), with a healthy subject heterozygous for the common F508del mutation, and with a healthy subject (no mutations) as internal control. The amplification product obtained from the subject carrying the 186-8T/C variant, using Exon 1–4 Forw/Rev primers, was undistinguishable from controls, moreover, using the exon2-spanning primer no amplification products were observed in the samples from any of the subjects (Negative Controls-NC). As control we amplified a portion (290 bp) of the housekeeping gene actin; this PCR was then carried out for 25 cycle.

We decided to investigate CFTR-channel function using NPD analysis and additional ex-vivo and molecular assays, aimed at supporting the previous findings, were carried out in parallel. Ferec et al. previously reported a possible exon 2 splicing in the genetic 186-13C > G variant detected in-trans with F508del in a 13 year old child with pancreatic sufficiency where nasal polyposis was the main symptom [14]. Although the T to C substitution is conservative, in this case we aimed to exclude the possibility that the genetic variant 186-8T/C could affect the splicing. For this reason, CFTR mRNA from blood cells was analyzed by RT-PCR, using exon 2 spanning primers or with primers designed to amplify fragments of different lengths depending on the presence or absence of exon 2 (Figure 3A). In all cases patient mRNA amplification products were identified as being the same as in a healthy donor (Figure 3B). Therefore we concluded that 186-8T/C mutation does not cause changes in the CFTR cDNA sequence despite its localization in intron 1 at position −8. However, the substitution of T with C is unlikely to be able to affect splicing since it is conservative in this polypyrimidine-rich, intronic region.

**Figure 4:**
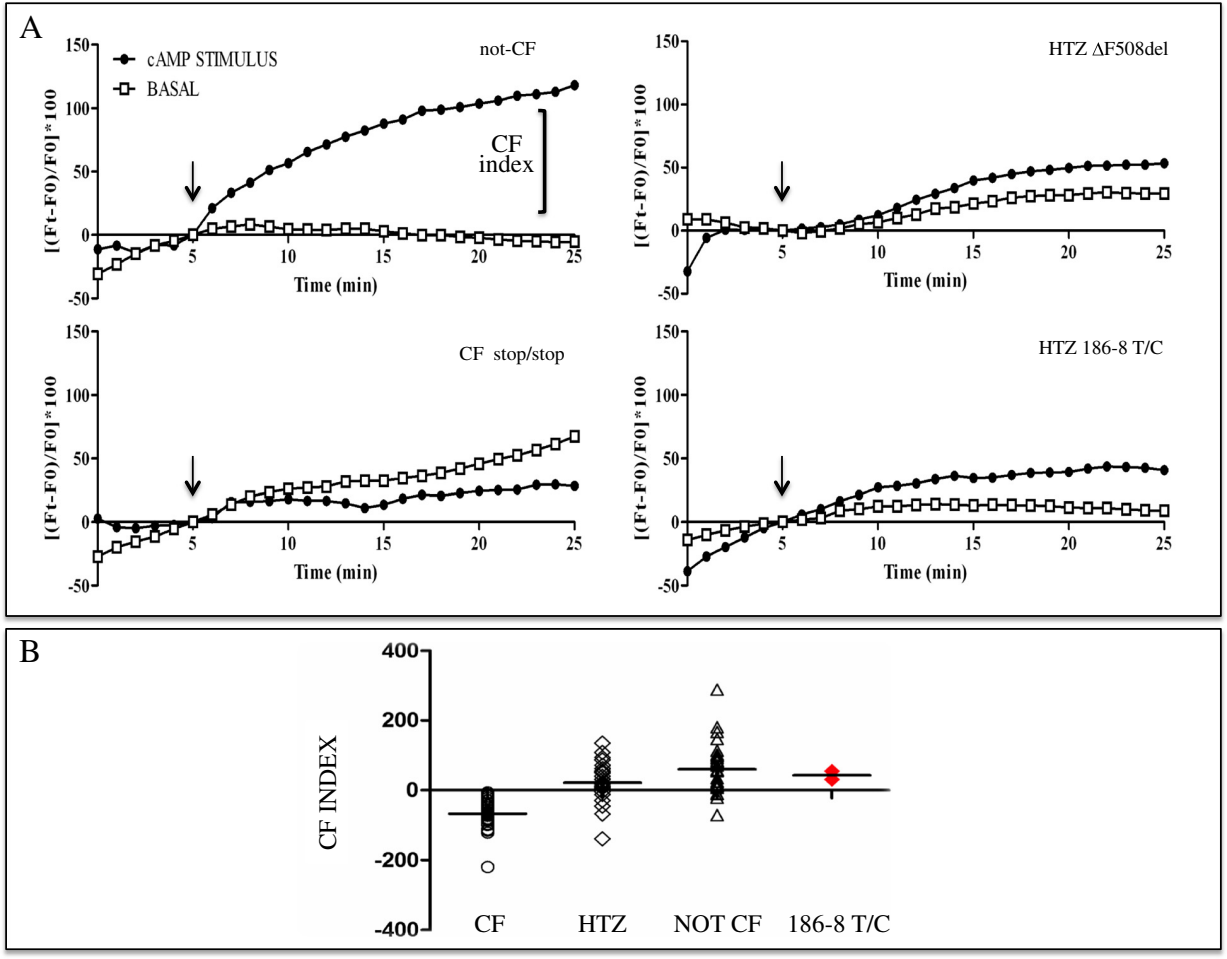
**Diagnostic algorithm. (A)***Monocyte cell membrane depolarization assay.* Single cell fluorescence assay was performed as described^10^. Measurements of the cell membrane depolarization were obtained in a extracellular environment lacking chloride-ion and were measured at two different conditions: under basal conditions (empty boxes), or with a stimulus-cocktail consisting of 8-Br-cAMP, Forkolin and IBMX (filled boxes) added at the 5th minute. Percentage of DiSBAC2(3) fluorescence variation (∆F) is shown over time according to the equation: ∆Ft = [(Ft-F0)/F0]*100, where Ft and F0 are the fluorescence values at time t and at the time t0 (5th minute). The difference between the maximum fluorescence values obtained on stimulation (filled boxes) and the matching fluorescence under basal conditions (empty boxes) represents the CF index. We defined the CF index outcome for this assay being a positive value in healthy subjects and a negative value in CF patients. A typical non-CF pattern, where a higher signal can be observed after stimulation, is represented at the top-left panel, with a CF index of +116. On the other hand, when monocytes from a subject carrying a non-sense mutation on both alleles were used, the opposite trend was observed (bottom-left panel), with a negative CF index of −25,2. With the monocytes from the patient (186-8T/C) a positive CF index was obtained (43 ± 12; n = 2; right-bottom panel), with a response to the cAMP-analogue similar to those obtained from the other HTZ subject (CF = +22; right-up panel). **(B)***CF index.* Plotting of the CF index values obtained from the subject in comparison with recording from patients and controls performed in our laboratory using the same experimental setting. Number of Samples: CF subjects n = 40, HTZ subjects n = 28 and non CF subjects n = 31. Analysis of the 186–8 T/C subject was repeated twice (technical duplicate).

Further CFTR protein function testing was carried out using single cell fluorescence analysis in monocytes. To summarize: the potential-sensitive probe bis-(1,3-diethylthiobarbituric acid) trimethine oxonol (DiSBAC2(3) Invitrogen, USA) was used to monitor CFTR-dependent membrane-potential (Vm) changes. We previously defined the CF index as an outcome of this assay and it was shown to be positive in healthy subjects and negative in CF patients [9]. In the genetic variant 186-8T/C the CF index was positive (mean: +43 ± 12; n = 2), a result that overlaps those obtained in monocytes/leukocytes from healthy donors (Figures 4A and 3B).

Doubts about the original CF diagnosis might also arise from the presence of interstitial infiltration in the lung in the absence of bronchiectasis and of CFTR dysfunction-causing mutations in this patient. Since at our center the patient had normal/ borderline sweat test values we did not investigate for eventual differential diagnosis with diseases associated with a raise in electrolyte levels [15].

In the case reported, the application at our Center of the CF diagnostic algorithm [4] was successful and the previous CF-diagnosis was not confirmed (Figure 1). Our interpretation of NPD data seems in agreement with the recommendation provided by Boucher [16] who underlined the importance to correctly evaluate sodium transport to establish a correct diagnosis when NPD are required. Based on a French study he proposed bronchiectasis to be a multiple hits process or a continuum of ion transport dysfunction measured by NPD according to presence of zero, one or two mutations or genetic variants. Diffuse bronchiectasis are a possible phenotype of CFTR related disorders. In this patients no bronchiectasis were evident by HRCT and low-level infiltrative interstitial lung damage in conjunction with recurrent bronchitis are still of unknown origin.

It is important to consider the negative impact of a wrong diagnosis of CF for a patient and relatives in terms of medical, psychological, social and insurance implications. It is indeed a chronic progressive disease compromising quality and expectancy of life. Considering its autosomal recessive inheritance also for genetic counselling it is very relevant to avoid wrong diagnosis, in particular in this case having no disease causing mutations identified.

This outlines how important is for the CF community to have access to clinical and electrophysiological information on rare mutations; it can lead to further improvements in diagnostic procedure and genetic counseling. A large number of genetic variants of uncertain functional significance have been identified due to the extensive sequencing being carried out, diluting the practical application of this valuable information [17,18]. Besides CFTR, several modifier genes have also been described as being able to influence CF phenotype [19]. CFTR functional tests are under development [20] but need to be validated and standardized assays are available only for NPD and intestinal current measurements [21]. Both these measurements are minimally invasive and can be used in selected cases.

## Conclusion

In the clinical case reported here, sweat tests and genetic analysis were inconclusive and CFTR functional testing, in accordance with a standard method (NPD), was carried out. In addition, we evaluated a possible exon skipping and CFTR function in leukocytes. None of the results obtained at our Center were consistent with CF diagnosis. An innovative approach using leukocytes was also applied, being potentially more convenient than currently available and standardized approaches. Validation with a large group of subjects is in progress and research to develop simpler, more feasible functional assays in leukocytes and epithelial cells are underway in our and in other Centres, with the aim of providing additional tools for testing genetic variants of uncertain clinical relevance as well as the effects of drugs targeting the basic CF defect.

## Consent

Written informed consent for publication of their clinical details and/or clinical images was obtained from the patient/parent/guardian/relative of the patient. A copy of the consent form is available for review by the Editor of this journal.

## Abbreviations

CF: Cystic Fibrosis; CFTR CF: Transmembrane Conductance Regulator gene; HRCT: High Resolution Computerized Tomography; NDP: Nasal potential difference; (DiSBAC2(3): bis-(1,3-diethylthiobarbituric acid) trimethine oxonol; BAL: Broncho Alveolar Lavage.

## Competing interests

The authors declare that they have no competing interests.

## Authors’ contributions

PM, and BA are clinicians responsible for the patient at the Cystic Fibrosis Center of Verona and had performed clinical trials. SC, GV, JJ, CA have performed the cellular studies. CS and MB read and commented the manuscript and are the responsible of the two laboratories that had performed these experiments. Authors read and approved the final manuscript.

## Pre-publication history

The pre-publication history for this paper can be accessed here:

http://www.biomedcentral.com/1471-2466/14/44/prepub
